# The WW-HECT protein Smurf2 interacts with the Docking Protein NEDD9/HEF1 for Aurora A activation

**DOI:** 10.1186/1747-1028-5-22

**Published:** 2010-09-08

**Authors:** Finola E Moore, Evan C Osmundson, Jennifer Koblinski, Elena Pugacheva, Erica A Golemis, Dipankar Ray, Hiroaki Kiyokawa

**Affiliations:** 1 Department of Molecular Pharmacology and Biological Chemistry, Northwestern University Feinberg School of Medicine, Chicago, IL, USA; 2 Department of Pathology, Northwestern University Feinberg School of Medicine, Chicago, IL, USA; 3 Robert H. Lurie Comprehensive Cancer Center, Northwestern University Feinberg School of Medicine, Chicago, IL, USA; 4 Department of Biochemistry, Mary Babb Randolph Cancer Center, West Virginia University; Morgantown, WV, USA; 5 Program in Molecular and Translational Medicine, Fox Chase Cancer Center, Philadelphia, PA USA; 6 Department of Radiation Oncology, University of Michigan Medical School, Ann Arbor, MI, USA

## Abstract

The multi-functional adaptor protein NEDD9/HEF1/Cas-L regulates cell motility, invasion and cell cycle progression, and plays key roles in cancer progression and metastasis. NEDD9 is localized to the centrosome and is required for activation of Aurora A kinase in mitosis. Here we demonstrate that the HECT-WW protein Smurf2 physically associates with NEDD9 and is required for the stability of NEDD9 protein. Smurf2 depletion results in a marked decrease in NEDD9 protein levels, by facilitating polyubiquitination and proteasomal degradation of NEDD9. Conversely, forced overexpression of Smurf2 results in upregulation of endogenous NEDD9 protein, confirming the role for Smurf2 in NEDD9 stability. Cells with Smurf2 depletion fail to activate Aurora A at the G_2_/M boundary, leading to a marked delay in mitotic entry. These observations suggest that the stable complex of Smurf2 and NEDD9 is required for timely entry into mitosis via Aurora A activation.

## Introduction

Smurf2 (Smad ubiquitination regulatory factor 2) is a HECT-E3 ligase that negatively regulates TGF-β signaling [1]. Smurf2 targets TGF-β type I receptor, Smad1, Smad2, Smad7, and the transcriptional repressor SnoN for degradation by the proteasome [1-4]. In addition to its role in TGF-β signaling, Smurf2 functions in diverse biological pathways, including those controlling the cell cycle and cell polarity/cytoskeletal remodeling [5-9]. Previous work from our laboratory demonstrated that Smurf2 protein levels vary during the cell cycle, peaking during mitosis [6]. The localization of Smurf2 also undergoes dynamic regulation. Smurf2 is at the centrosome from G_1 _through prophase, then localizes to the spindle midzone during anaphase, and the midbody during cytokinesis [6]. To date, the best-defined role of Smurf2 in mitosis involves its binding to and stabilization of Mad2, which is required for the spindle assembly checkpoint [6].

Smurf2 contains WW domains, which mediate interactions with proteins that have PPxY motifs [10], while Mad2 does not possess any PPxY motif, suggesting other mitosis-relevant partners might exist for Smurf2. For further insight into the cell cycle-regulatory role of Smurf2, we used a candidate-based approach to select for potential Smurf2 interactors, examining those proteins that both contain a PPxY-motif and exhibit a similar subcellular localization pattern. NEDD9 (neural precursor cell expressed, developmentally down-regulated 9, also called HEF1, human enhancer of filamentation 1 and Cas-L Crk-associated substrate related, lymphocyte-type) is a scaffold protein that contains a PPxY motif [11]. NEDD9 displays similar protein expression and localization pattern as Smurf2, rising in G_2 _and decreasing after mitosis, localizing to the centrosome, midzone, and midbody [12]. The localization of NEDD9 to the centrosome is required for proper mitotic entry [12]. The cell cycle-regulatory function of NEDD9 is mediated, at least partly, by its role for the activation of Aurora A kinase. Centrosomal Aurora A activity is a critical step for mitotic entry from the G_2 _phase, required for the initial activation of Cyclin B-CDK1 at the centrosome [13]. Among the elements recruited to the centrosome at the G_2_/M boundary are the activators of Aurora A, such as Ajuba, TPX2 and NEDD9. Thus, NEDD9 plays a significant role in triggering coordinated activation of the mitotic kinase cascade from Aurora A to Cyclin B-CDK1 and perhaps other mitotic kinases required for proper progression of mitosis [14].

To date, the upstream mechanisms that control the level of NEDD9 protein during mitotic progression have been poorly understood. Here we demonstrate that Smurf2 regulates NEDD9 levels by preventing its proteasomal degradation and this control is rate-limiting for Aurora A activation and mitotic entry. Our data indicate a novel regulatory pathway critical for timely mitotic entry.

## Results

### Smurf2 and NEDD9 interact

**Figure 1:**
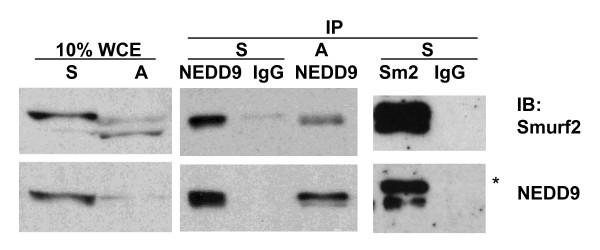
**Smurf2 physically interacts with NEDD9 during mitosis**. Extracts were prepared from HeLa cells that were either asynchronous (A) or synchronized at mitosis by the thymidine-nocodazole protocol (S), and 400 μg proteins were immunoprecipitated (IP) with the indicated antibodies or normal immunoglobulin (IgG), followed by immunoblotting (IB) for the indicated proteins. Forty micrograms of whole cell extracts (10% WCE) were also subjected to IB for comparison.

Smurf2 contains WW domains that confer interaction with PPxY motifs [1,15]. Our previous finding that Smurf2 localizes to the centrosome and is required for proper mitotic progression [6] prompted us to examine whether Smurf2 physically interacts with other proteins known to regulate mitosis at the centrosome. Database analyses identified several centrosomal proteins that contain PPxY motifs, including Centriolin, LATS1, LATS2 and NEDD9. Since NEDD9 plays diverse roles not only in focal adhesion and cell motility but also in mitotic regulation, we further analyzed the potential interaction between Smurf2 and NEDD9. NEDD9 contains a PPxY motif at residues 108-111, YQVPPSYQNQ, within an SH2 binding site-rich domain. The protein levels of both Smurf2 and NEDD9 rise during late G_2 _and significantly decline exiting mitosis [6,16]. To maximize the potential of finding a physical interaction, HeLa cells were synchronized in mitosis by nocodazole treatment and then lysates were prepared for co-immunoprecipitation assays. Indeed, Smurf2 was readily detected in NEDD9 immunoprecipitates from mitotic cells, and reciprocally, NEDD9 was found in Smurf2 immunoprecipitates (Figure 1). Co-immunoprecipitation of these two proteins was less obvious in an asynchronous cell population. Our data do not exclude that this interaction could require other proteins, but these data do suggest that Smurf2 and NEDD9 are in complex with each other, most abundantly so during mitosis.

### Depletion of Smurf2 destabilizes NEDD9

**Figure 2:**
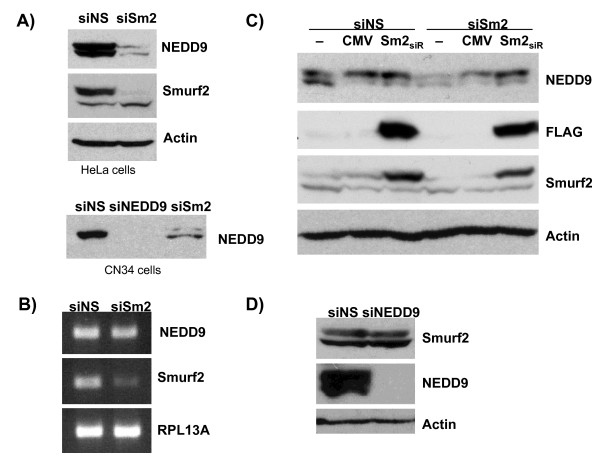
**Depletion of Smurf2 leads to a decline in NEDD9 protein levels**. (A) Depletion of Smurf2 by siRNA (siSm2) leads to decreased NEDD9 protein levels in HeLa and CN34 cells. (B) Smurf2 does not affect NEDD9 transcript level. RNA samples from HeLa cells transfected with siSmurf2 were analyzed by RT-PCR for the indicated transcripts. (C) Forced expression of an siRNA-resistant Smurf2 mutant (Sm2_siR_) rescues NEDD9 levels in HeLa cells transfected with siSmurf2. D) NEDD9 depletion does not affect Smurf2 levels. For all samples, protein from HeLa cells was harvested 48 hours after transfection, 30 μg total protein was loaded onto gel. For siRNA transfections, cells were reverse transfected with 50 nM siRNA (siSmurf2#1, siNEDD9 Smartpool). RT-PCR was performed on cells treated as described above. RNA was extracted as described in Methods. For siRNA and DNA transfection, cells were forward transfected with 50 nM siRNA and 1 μg DNA of either no plasmid, CMV empty vector, FLAG-Smurf2_si-resistant mutant_.

To begin to examine the functional significance of the Smurf2-NEDD9 interaction, we tested whether Smurf2 affected the level of NEDD9. In particular, we addressed whether the E3 ligase Smurf2 would directly target NEDD9 for proteasome-mediated degradation. Contrary to this speculation, it was consistently observed that NEDD9 protein levels were decreased by siRNA-mediated Smurf2 depletion in HeLa cervical carcinoma cells and CN34 mammary carcinoma cells (Figure 2A), and U2OS osteosarcoma cells (data not shown). The decline in NEDD9 protein levels induced by Smurf2 depletion was a post-transcriptional effect as RT-PCR analysis showed little to no effect on NEDD9 mRNA level (Figure 2B). To exclude the possibility of off-target effects exerted by Smurf2 siRNA, we engineered a Smurf2 mutant (FLAG-Smurf2_si-resistant_) that is resistant to siRNA-mediated knockdown by mutating 4 nucleotides within the siSmurf2#1 target region. Co-transfection of siSmurf2-treated cells with FLAG-Smurf2_si-resistant _mutant significantly restored NEDD9 levels (Figure 2C). These results show that Smurf2 plays an essential role in maintaining the stability of NEDD9 protein. To reciprocally establish whether NEDD9 controls Smurf2 levels, HeLa cells were transfected with NEDD9 siRNA. NEDD9 depletion had no discernable effect on Smurf2 protein levels (Figure 2D). These data suggest that Smurf2 positively regulates the level of NEDD9 protein at the post-transcriptional level.

### Regulation of NEDD9 by Smurf2 is mediated by the proteasome

**Figure 3:**
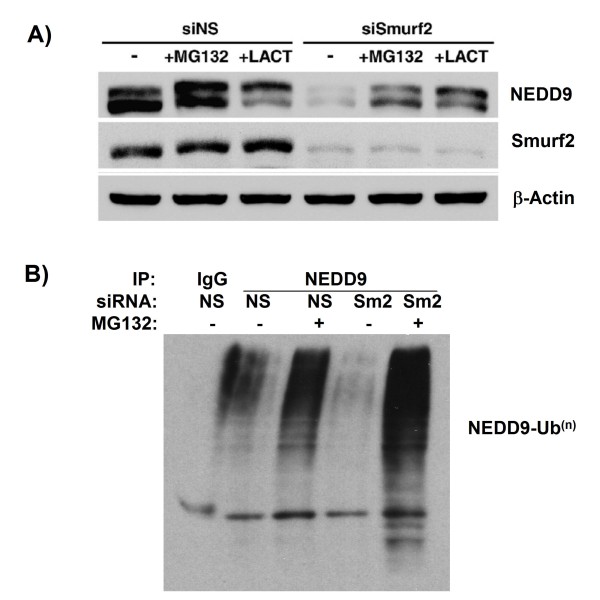
**Depletion of Smurf2 results in enhanced polyubiquitination and proteasomal degradation**. (A) Decline in NEDD9 induced by siSmurf2 (Sm2) was rescued by treatment with proteasomal inhibitors. siRNA-transfected HeLa cells were treated with either 2 μM MG132, 5 μM lactacystin (LACT) or equivalent volume DMSO for 4 h. (B) siSmurf2 leads to polyubiquitination of NEDD9. NEDD9 was immunoprecipitated from HeLa cells transfected with siRNA and treated with MG132.

Since NEDD9 is known to undergo proteasomal degradation [17], we next examined whether Smurf2 depletion accelerates this process (Figure 3A). As we expected, the proteasome inhibitors MG132 and lactacystin substantially restored NEDD9 protein levels in cells with Smurf2 depleted (Figure 3A). To determine whether the proteasomal degradation of NEDD9 was dependent on polyubiquitination, NEDD9 was immunoprecipitated from Smurf2-depleted HeLa cells treated with MG132, and then analyzed by immunoblotting with anti-ubiquitin antibody (Figure 3B). Polyubiquitination of NEDD9 was significantly enhanced in cells treated with Smurf2 siRNA and MG132, compared with that in cells with non-specific siRNAs and MG132. These results suggest that depletion of Smurf2 leads to polyubiquitination and subsequent degradation of NEDD9.

### Overexpression of Smurf2 stabilizes NEDD9 in a ligase-independent manner

**Figure 4:**
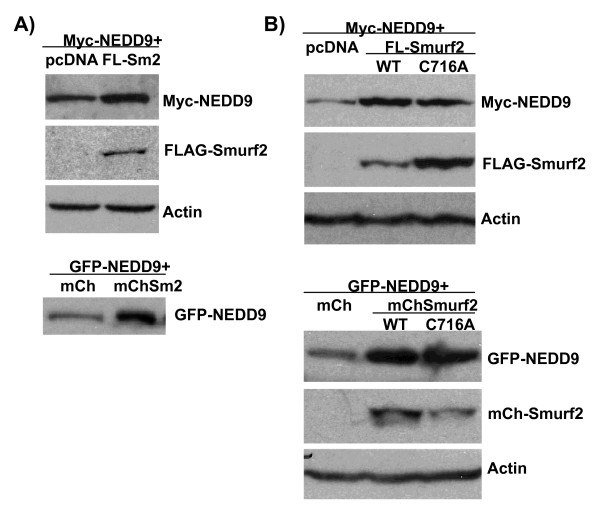
**Forced expression of wild-type or catalytically inactive Smurf2 stabilizes NEDD9 protein**. (A) HeLa cells were co-transfected with Myc-NEDD9 and either empty vector (pcDNA) or FLAG-Smurf2 (Sm2). Similar conditions were used to co-transfect with GFP-NEDD9 and either empty vector (mCherry) or mCherry-Smurf2. After 48 h, cells were harvested for immunoblotting. (B) HeLa cells were co-transfected with the indicated plasmid as described in (A). Co-transfection of wild-type (WT) or catalytically inactive (C716A) Smurf2 increased NEDD9 expression.

We next asked if forced overexpression of Smurf2 increased NEDD9 levels. HeLa cells were co-transfected with Myc- or GFP-tagged NEDD9 and FLAG- or mCherry-tagged Smurf2, and then analyzed by immunoblotting for each tag (Figure 4). Levels of Myc- or GFP-tagged NEDD9 were substantially increased by co-transfection with FLAG- or mCherry-tagged Smurf2, compared with plasmid controls. The upregulation of NEDD9 by co-transfection with Smurf2 was not a by-product of altered cell cycle progression, because flow cytometric analysis after transfection with GFP-NEDD9 and mCherry-Smurf2 showed that the percentages of transfected cells in G_1_, S and G_2_/M were not significantly affected by Smurf2 co-transfection at the time points examined (data not shown). As Smurf2 is an E3 ligase, we next asked if the Smurf2 enzymatic activity was required for it to affect NEDD9 expression. Interestingly, co-expression of a catalytically inactive Smurf2 mutant (C716A, targeting the HECT domain) also upregulated the levels of exogenously expressed NEDD9. These results provide support for the idea that Smurf2 positively regulates NEDD9, and suggest that this action may be independent of the activity of Smurf2 as an E3 ligase.

### Smurf2 depletion results in delayed mitotic entry with impaired Aurora A activation

**Figure 5:**
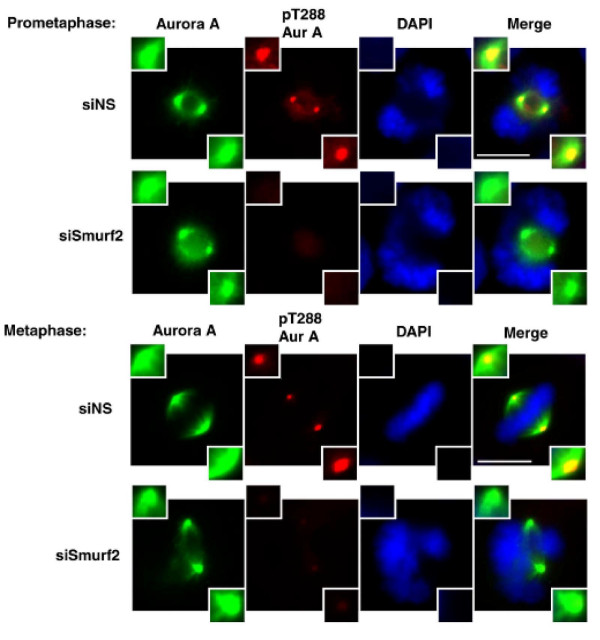
**Depletion of Smurf2 inhibits Aurora A activation at the centrosome**. HeLa cells were transfected with control non-specific dsRNAs (siNS) or siRNA against Smurf2. At 40 h post-transfection, cells were fixed, stained with DAPI and pan-Aurora A or Thr288 phosphorylation-specific (pT288) Aurora A antibody, and subjected to immunofluorescence microscopy. Smurf2-depleted cells exhibit a marked reduction in Thr288 phosphorylated (active) Aurora A at the spindle poles.

**Figure 6:**
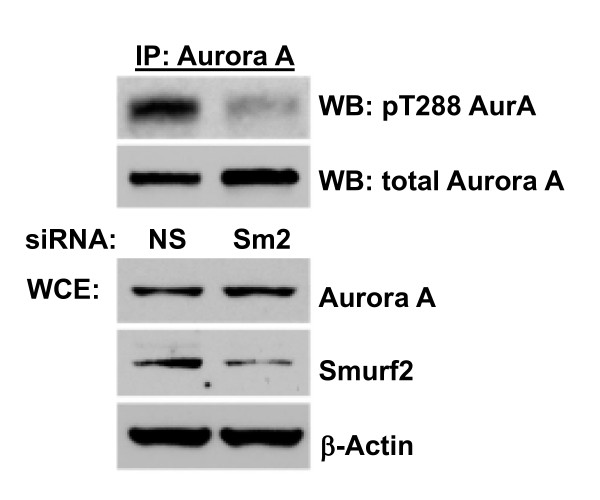
**Depletion of Smurf2 decreases the activated pool of Aurora A**. Lysates of control and Smurf2 depleted HeLa cells were first immunoprecipitated with pan-Aurora A antibody and then western blots were probed with antibody specific for Thr288 phosphorylated active Aurora-A.

We recently demonstrated that Smurf2 regulates the spindle assembly checkpoint during early to mid-mitosis, via action controlling the stability of Mad2 protein [6]. HeLa cells depleted of Smurf2 exhibit perturbed chromosome segregation, premature anaphase onset, and the inability to arrest in prometaphase in response to nocodazole or taxol. The finding that Smurf2 controls NEDD9 levels prompted us to examine the impact of Smurf2 depletion on mitotic entry, as NEDD9 has been demonstrated to be required for timely entry into mitosis with proper Aurora A activation [14]. HeLa cells transfected with Smurf2 siRNA, NEDD9 siRNA or control siRNAs were synchronized at early S by double thymidine block [6], released into synchronous cell cycle progression and analyzed at prometaphase and metaphase (Figure 5). Immunofluorescence microscopy showed that Thr288 phosphorylation of Aurora A at centrosomes, reflecting auto-phosphorylation and activation, was significantly diminished to almost undetectable levels in mitotic cells with Smurf2 depletion, relative to control cells at the same time point. Immunoprecipitation followed by immunoblotting detection of Thr288 phosphorylation separately confirmed that Smurf2 depletion reduced Aurora A activation (Figure 6).

**Figure 7:**
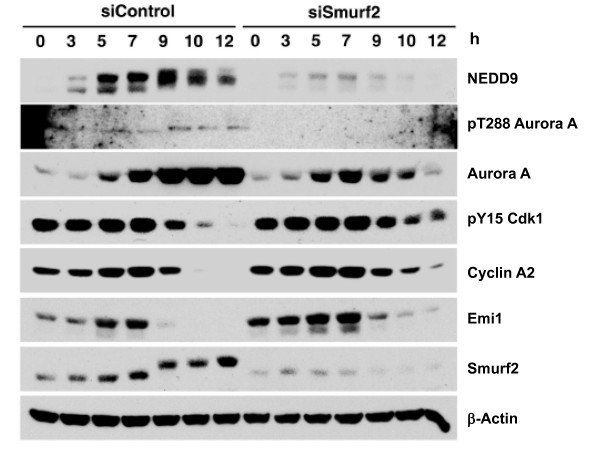
**Depletion of Smurf2 results in the lack of Aurora A activation and delayed mitotic entry**. HeLa cells were transfected with siRNA against Smurf2 or control dsRNAs (siNS), synchronized at early S phase by a double thymidine protocol, and released into synchronized progression toward mitosis. Cells were harvested at the indicated times after release for immunoblotting with the antibodies shown on right.

To determine whether these results reflected a specific requirement of Smurf2 for Aurora A activation or a general effect on mitotic signaling, we performed immunoblotting for representative mitosis-regulatory proteins at 0-12 hours after release from thymidine-induced S phase arrest (Figure 7). In control cells, the active form of Aurora A with Thr288 phosphorylation appeared around 8-9 hours after release, which coincided with or was followed by downregulation of Cyclin A and a migration shift of Smurf2 due to mitosis-specific phosphorylation. Further, CDK1 inhibition as measured by phosphorylation at Tyr15 is relieved beginning 9 hours post-release. Aurora A activation was substantially diminished in cells transfected with NEDD9 siRNA, as demonstrated previously [14]. Similar diminishment in Thr288 phosphorylation of Aurora A was observed in cells transfected with Smurf2 siRNA, which also displayed a significant decrease in NEDD9 levels. Consistently, downregulation of Cyclin A2 and Emi1 and the mitosis-associated shift in Smurf2 migration were delayed in cells with NEDD9 depletion or Smurf2 depletion. In addition, the master mitotic regulator CDK1 remained in its inhibited, Tyr15-phosphorylated form longer in cells depleted of Smurf2. Taken together, these data suggest that Smurf2 is a determining factor of NEDD9 levels and Aurora A activation in mitotic cells.

## Discussion

In the present study we have demonstrated the novel regulation of the multi-functional scaffold protein NEDD9 by the WW-HECT protein Smurf2. Physical interaction with Smurf2 leads to stabilization of NEDD9 protein via suppression of polyubiquitination and subsequent proteasomal degradation. Interestingly, stabilization does not appear to depend on the E3 ligase activity of Smurf2. Depletion of Smurf2, as well as NEDD9 depletion, results in impaired activation of Aurora A at the G_2_/M boundary. These results support the notion that Smurf2 is a critical regulator of entry into mitosis, extending our recent study on the role for Smurf2 in Mad2 regulation and the Spindle Assembly Checkpoint.

Aurora A activation during late G_2 _is a critical step for commitment to mitosis, and prerequisite for proper activation of Cyclin B-CDK1 and other mitotic kinases [13]. Centrosomal Aurora A activity governs the timing of mitotic entry, triggering nuclear envelop breakdown at prophase [18]. Recent studies demonstrated the requirement for NEDD9 in Aurora A activation and suggested that this scaffold protein is a critical component of mitosis regulation [12,14]. NEDD9 expression is regulated in a cell cycle-dependent manner and peaks in G_2 _and M, when it accumulates at the centrosome together with Aurora A. NEDD9 together with other Aurora A activators such as TPX2 and Ajuba stimulates autophosphorylation of Aurora A at Thr288, which is required for full activation of the kinase. Aurora A then phosphorylates NEDD9 at Ser296, leading to dissociation of the complex and allowing Aurora A to interact with other substrates. Our finding that Smurf2 promotes Aurora A activation does not exclude possible effects of Smurf2 on other Aurora A regulators such as TPX2 and Ajuba. The mitotic function of NEDD9 could be related to its key role in focal adhesion-dependent migration [reviewed in [19,20]]. NEDD9 associates with focal adhesion kinase (FAK) and a Src family kinase. Subsequent Src-mediated phosphorylation of NEDD9 creates active SH2 sites, which bind to the adaptor protein Crk. Crk association subsequently recruits DOCK180 and C3G, eliciting signals to the GTPases Rac and Rap, respectively. A number of recent studies suggested the presence of crosstalk between the focal adhesion attachment signaling and the centrosome-based mitosis signaling [21-23]. Multiple components of integrin-mediated migratory signaling including NEDD9 and Pak have been shown to associate with and activate Aurora A at the centrosome. Another centrosomal protein GIT1, which is required for Pak localization to the centrosome, binds to the focal adhesion protein Paxillin. Furthermore, the mitotic LATS1 kinase in complex with the focal adhesion protein Zyxin localizes to microtubules proximal to the centrosome and regulates mitotic initiation [24]. It is noteworthy that LATS1 also possesses a PPxY motif for potential association with the WW domains of Smurf2, although its significance remains to be determined.

Smurf2 also plays multiple roles in cell migration and mitotic regulation [5,25]. Among the substrates for Smurf2-mediated polyubiquitination are TGF-β type 1 receptor, the GTPase Rap1B, and its closely related homolog, Smurf1 [1,8,26]. Smurf1 polyubiquitinates RhoA, talin head domain and hPEM2 [27,28]. These proteins are all involved in the control of cell migration. Moreover, a recent study demonstrated that Smurf2 and Smurf1 are critical regulators of planar cell polarity. Mice deficient for *Smurf1 *and *Smurf2 *display defects in planar cell polarity that leads to perturbed stereocilia alignment in neurosensory cells of the cochlea and failed closure of the neural tube [7]. Our recent work provided evidence that Smurf2 is also a regulator of mitosis [6]. Smurf2 expression fluctuates during the cell cycle, with a peak around the G_2_/M boundary. Smurf2 localizes to the centrosome from interphase until late mitosis, when it moves to the mitotic midbody together with the chromosome passenger complex. Smurf2-depleted cells exhibit multiple defects associated with impaired spindle assembly checkpoint such as premature activation of the anaphase promoting complex (APC) in prometaphase, misaligned and lagging chromosomes during the metaphase to anaphase transition, and failed cytokinesis. These defects are attributable partly to a marked decrease in the spindle checkpoint protein Mad2, as a consequence of accelerated proteasomal degradation. The present study demonstrates that Smurf2 depletion also downregulates NEDD9, which results in impaired Aurora A activation and delayed mitotic entry. The integrin signaling including NEDD9, which governs the basal cell adhesion to the extracellular matrix, determines the orientation of the cell division plane together with the cadherin-mediated planar adhesion signaling. Thus, the crosstalk involving Smurf2, NEDD9 and Aurora A may function as effectors of attachment-sensing mitotic checkpoint. Also, Smurf2 and NEDD9 may collaborate in RhoA activation critical for not only migration but also cytokinesis [26,29]. Taken together, these data imply that in proliferating cell types Smurf2 controls various protein complexes that are critical for different phases of mitosis, i.e., the NEDD9-Aurora A centrosomal complex in G_2 _and prophase, the Mad2 spindle checkpoint complex in prometaphase, and the RhoA complex in cytokinesis. Since Smurf2 is known to play diverse roles in the biology of non-proliferative differentiated cells, it will be important to determine whether the mitosis-promoting function of Smurf2 is one of cell type-specific events or a more conserved mechanism of proliferation.

The mechanism with which Smurf2 controls NEDD9 stability remains to be elucidated. The stability of NEDD9 protein is regulated by phosphorylation and subsequent polyubiquitination [30]. In response to TGF-β signals, NEDD9 undergoes polyubiquitination facilitated by physical interaction with Smad3 [17,31]. Additionally, another member of the WW-HECT family, AIP4 (atrophin 1 interacting protein 4)/Itch, can also target NEDD9 for degradation in a TGF-β-dependent manner [32]. Further, APC/C^Cdh1 ^targets NEDD9 for degradation at the end of mitosis [31]. We found that phosphorylated and hyperphosphorylated NEDD9 are stabilized by Smurf2. Though Smurf2 is known as a negative regulator of TGF-β signaling, the NEDD9-stabilizing action of Smurf2 seems unlikely to depend on altered TGF-β signaling. HeLa cells are not typically responsive to TGF-β signals [33]. Further, we found that depletion of Smad3, Smurf1, or AIP4/Itch failed to rescue NEDD9 levels in cells with Smurf2 depletion (data not shown). We believe that the Smurf2 regulation of NEDD9 in mitotic entry occurs through a different mechanism from Smurf2 regulation of Mad2 in the Spindle Assembly Checkpoint. It is likely that Smurf2 interacts with Mad2 and NEDD9 at distinct subcellular locations during mitosis. At the kinetochore and its proximity, Smurf2 may target an intermediary E3 ligase for degradation to stabilize Mad2. In contrast, Smurf2 at the centrosome binds and stabilizes NEDD9 apparently in a ligase-independent fashion. Currently several hypotheses are being evaluated regarding NEDD9 stabilization by Smurf2. Our observation that the catalytically inactive mutant of Smurf2 could also stabilize NEDD9 levels excludes the possibility that Smurf2 targets an intermediary ligase for NEDD9 degradation. Consistent with the ligase-independent function of Smurf2 is a previous report that overexpression of wild-type or ligase-dead Smurf2 induces senescence [34]. Further, AIP4/Itch stabilizes Smad7/TGFβRI complex independently of its ligase activity [35]. Smurf2 also interacts with Smad7, and does not immediately induce its degradation [1]. Interestingly, NEDD9 has been shown to interact with Smad7 [36]. These data also exclude a model in which NEDD9 is stabilized by monoubiquitination. Smurf2 may sequester NEDD9 away from locations in the cell where it could encounter its E3 ligase. Alternatively, Smurf2 may instead mask regulatory epitopes for ubiquitination. Smurf2 may serve as an adaptor for an unidentified regulator that counteracts with another E3 ligase promoting NEDD9 degradation. The ongoing studies are expected to identify the E3 ligase that targets NEDD9 for degradation in response to Smurf2 depletion, and to reveal missing components of the Smurf2-dependent mitosis-regulatory pathway.

Both Smurf2 and NEDD9 are overexpressed in multiple types of cancers. Smurf2 upregulation has been associated with poor prognosis in cancers including esophageal squamous cell carcinoma and renal cell carcinoma [37,38]. Smurf2 has also been found to be upregulated in breast cancer tissue and cell lines as well as ovarian and prostate cancer cell lines [39]. Jin and colleagues found that depletion of Smurf2 by siRNA inhibited migration and invasion, overexpression of Smurf2 led to enhanced migration and invasion [39]. Together, these data suggest that Smurf2 promotes tumor cell migration and invasion. Increased levels of NEDD9 have been found in lung adenocarcinoma [40], glioblastoma [41], and melanoma [42]. NEDD9 was identified as one of a few critical genes that mediate metastasis in melanoma [42] and breast cancer [43]. Mice null for *Nedd9 *are resistant to MMTV-polyoma T-induced tumorigenesis [40], recapitulating the significant role for NEDD9 in tumor development. It will be important to determine whether Smurf2 and NEDD9 levels correlate with each other in human cancers. Future studies using human cancer specimens should provide insight into the putative oncogenic interaction of these two proteins in the regulation of cell cycle progression and genomic instability of cancer cells.

## Conclusion

The present work demonstrates that Smurf2 positively regulates NEDD9, which is required for Aurora A activation and proper mitotic entry. These data suggest that Smurf2 plays diverse roles in mitotic regulation.

## Methods

### Cell lines and reagents

HeLa human cervical carcinoma cells (ATCC) were cultures under standard conditions of complete medium containing DMEM, 10% fetal bovine serum (FBS), 2 mM glutamine, 100 units/ml Penicillin/Streptomycin. CN34 breast cancer cells were cultured as described in [44]. Antibodies used in this study are NEDD9/HEF1/Cas-L (2G9), Ubiquitin (P4D1), normal rabbit IgG, normal mouse IgG from Santa Cruz Biotechnology (Santa Cruz, California); Smurf2 from Upstate/Millipore (Lake Placid, NY); FLAG M2 and β-Actin (Clone AC-15) from Sigma Aldrich; and Myc from Invitrogen.

### Plasmids and siRNA reagents

siRNA was ordered from Dharmacon/Thermo Fisher Scientific for Smurf2 (#1), NEDD9 (smartpool) and control (#3). The sequence for siSm2 was 5'-GAUGAGAACACUCCAAUUAUU-3'. NEDD9 and its mutants were sub-cloned into Myc vector from Sigma Aldrich. FLAG-Smurf2WT and FLAG-Smurf2(C716A) in pCs2+ vector were kindly provided by Gerald Thomsen at Stony Brook University. Smurf2 was sub-cloned into mCherry vector from Clontech. Smurf2_si-resistant _was created by site-directed mutagenesis (Quickchange from Stratagene) of FLAG-Smurf2 at 4 nucleotides within the region targeted by siSmurf2#1: T631C, G634A, T640G, A643G. For protein and RNA extractions, cells were reverse transfected with 50 nM siRNA using RNAiMax from Invitrogen, then harvested 48 h later. When DNA was transfected, 1 μg of each plasmid per 6D dish was transfected with Lipofectamine2000 from Invitrogen.

### Co-immunoprecipitation

For immunoblotting or immunoprecipitation, cells were lysted by sonication in lysis buffer as described previously [6]. Unless otherwise noted, 30 μg total protein lysate was loaded onto gel. Co-immunoprecipitation was performed in HeLa cells that were either asynchronous synchronized at mitosis by 2 mM thymidine 18 h, release for 9 h, 400 ng/μl nocodazole for 14 h. 400 μg total protein was incubated with 1.5 μg of antibody overnight at 4°C. Protein A (for rabbit Smurf2 IPs) or G (for mouse NEDD9 IPs, both from Zymogen) beads were added for 1 h 4°C. Immunoprecipitated materials were loaded onto 2 different gels and probed by Western blot accordingly. For immunoprecipitation with NEDD9 antibody, lysates were pre-cleared with protein G beads for 30 minutes 4°C before incubation with NEDD9 antibody. For NEDD9 immunoprecipitation for ubiquitination, cells were treated for 4 h with 2 μM MG132 44 h post-transfection. Entire immunoprecipitates were loaded onto one gel, gel was transferred onto PVDF membrane as usual, then prepared for Ubiquitin blotting by treatment with 6 M guanidium chloride, 20 mM Tris pH 7.5, 1 mM PMSF (fresh), 5 mM β-mercaptoethanol (fresh) for 30 minutes, 4°C.

### Semi-quantitative RT-PCR

RNA was extracted with Agilent kit, 2 μg RNA was used to synthesize cDNA with the Invitrogen Superscript II kit, PCR was performed with 2 μl of cDNA, 27 cycles, T_m _or 50°C.

### Immunofluorescence

HeLa cells were grown on coverslips and fixed in ice cold methanol for 20 minutes to overnight. Centrosome staining was followed as described previously [6].

## Abbreviations

NEDD9: Neural precursor cell expressed, developmentally down-regulated 9, also called HEF1: Human enhancer of filamentation 1, and Cas-L: Crk-associated substrate related, lymphocyte-type; TGF-β: Transforming growth factor; TPX2: Target protein for *Xenopus *kinesin-like protein 2; CDK1: Cyclin dependent kinase 1; LATS1, LATS2: Large tumor suppressor; GFP: green fluorescent protein; siRNA: short interfering RNA

## Competing interests

The authors declare that they have no competing interests.

## Authors' contributions

FM and ECO performed the experiments shown here. FM carried out the immunoblotting, RT-PCR, immunoprecipitation, plasmid cloning, and flow-cytometry discussed in Figures 1, 2, 3, &4. ECO performed immunoblotting shown in Figure 3, immunofluorescence of Figure 5, immunoprecipitation and immunoblotting for Figures 6 and 7. FM, ECO, JK, DR, and HK participated in the design and coordination of the study. In particular, ECO, DR and HK initiated this project from the phase of substantiating its concept; FM, DR, and HK completed the project up to the phase of writing the manuscript. EP and EG contributed to the design of this study, providing scientific and technical advices and critical reagents for this study. All authors read and approved the final manuscript.
